# Scaffold size-dependent effect on the enhanced uptake of antibiotics and other compounds by *Escherichia coli* and *Pseudomonas aeruginosa*

**DOI:** 10.1038/s41598-022-09635-6

**Published:** 2022-04-04

**Authors:** Kyosuke Yamamoto, Nao Yamamoto, Shotaro Ayukawa, Yoshiaki Yasutake, Koji Ishiya, Nobutaka Nakashima

**Affiliations:** 1 Bioproduction Research Institute, National Institute of Advanced Industrial Science and Technology (AIST), Toyohira-ku, Sapporo, 062-8517 Japan; 2 School of Life Science and Technology, Tokyo Institute of Technology, 2-12-1-M6-5 Ookayama, Meguro-ku, Tokyo, 152-8550 Japan; 3 Computational Bio Big-Data Open Innovation Laboratory (CBBD-OIL), AIST, Tokyo, 169-8555 Japan

**Keywords:** Microbiology, Biotechnology

## Abstract

The outer membrane of Gram-negative bacteria functions as an impermeable barrier to foreign compounds. Thus, modulating membrane transport can contribute to improving susceptibility to antibiotics and efficiency of bioproduction reactions. In this study, the cellular uptake of hydrophobic and large-scaffold antibiotics and other compounds in Gram-negative bacteria was investigated by modulating the homolog expression of *bamB* encoding an outer membrane lipoprotein and *tolC* encoding an outer membrane efflux protein via gene deletion and gene silencing. The potential of deletion mutants for biotechnological applications, such as drug screening and bioproduction, was also demonstrated. Instead of being subjected to gene deletion, wild-type bacterial cells were treated with cell-penetrating peptide conjugates of a peptide nucleic acid (CPP-PNA) against *bamB* and *tolC* homologs as antisense agents. Results revealed that the single deletion of *bamB* and *tolC* in *Escherichia coli* increased the uptake of large- and small-scaffold hydrophobic compounds, respectively. A *bamB*-and-*tolC* double deletion mutant had a higher uptake efficiency for certain antibiotics and other compounds with high hydrophobicity than each single deletion mutant. The CPP-PNA treated *E. coli* and *Pseudomonas aeruginosa* cells showed high sensitivity to various antibiotics. Therefore, these gene deletion and silencing approaches can be utilized in therapeutic and biotechnological fields.

## Introduction

Gram-negative bacteria have inner and outer membranes, and their outer membrane functions as an impermeable barrier to foreign compounds. Compounds > 600 Da in size are not internalized into the cells of Gram-negative bacteria via passive diffusion^1,2^. Bacteria use various membrane pumps to actively transport compounds across cell membranes. Therefore, the uptake efficiency of foreign compounds is determined by the ratio of influx and efflux through membranes mediated by passive diffusion and active transportation; uptake efficiency has been modified from medical and biotechnological viewpoints^3^.

As many antibiotics work inside their target cells, shutting off their entry into cells and pumping them out of cells are common antimicrobial resistance mechanisms of bacteria^4^. Several mutants of *Escherichia coli* have been isolated to explore ways on how to improve the uptake efficiency of antimicrobial compounds. Mutations in *lpx* involved in lipid A synthesis decrease the minimum inhibitory concentration (MIC) of antibiotics such as rifampicin, erythromycin, and fusidic acid^5^. In a comprehensive analysis involving a single-gene deletion collection, a deletion mutant of *bamB* (encoding the outer membrane lipoprotein), *tolC* (encoding the outer membrane efflux protein), *waaC* (participating in lipopolysaccharide biosynthesis), or other cell membrane-related genes has a decreased MIC of various antibiotics^6^. In many biotechnological applications, bacterial cells are required to uptake foreign compounds and maintain high intracellular concentrations for intracellular reactions. Therefore, the improvement of the substrate uptake rate of microbial cells (e.g., transport engineering) is a key approach for efficient industrial bioproduction^3,7^. For example, *tolC* deletion mutants of *E. coli* have a relatively high uptake of cholesterol derivatives such as vitamin D3 (VD3)^8^. Although previous studies identified key genes for the improvement of uptake efficiency, the combined effects of these genes have been rarely evaluated and should be further improved.

Various approaches other than gene deletion can increase uptake efficiency; for example, low-molecular-weight sensitizers, along with target compounds, can be used to increase the permeability of cell membranes. One of the most used sensitizers for increasing the membrane permeability of Gram-negative bacteria is polymyxin B nonapeptide, which decreases the MIC of various antibiotics^9^. Moreover, MAC13243, which inhibits periplasmic chaperone protein LolA activity, sensitizes cells to large-scaffold antibiotics^1^. Although these sensitizer compounds are generally useful and easy to handle, it is still difficult and laborious to design or find ideal sensitizers having expected effects without causing side effects (e.g., cell toxicity).

In this study, we applied gene deletion and sensitizing approaches to increase the uptake efficiency of *E. coli* and investigated the influx and efflux properties of several compounds in terms of their hydrophobicity and scaffold size. We selected genes to be targeted according to the following criteria: (1) complete deletion of a chosen gene does not cause severe growth arrest or retardation, (2) the gene is conserved among Gram-negative bacteria, and (3) multiple target genes may have different cellular functions that contrast with one another for investigating the uptake efficiency associated with multiple cellular functions. Based on these criteria, we chose *bamB* and *tolC*. The null mutants of both genes are not associated with obvious growth defects^8,10^, and these genes are well conserved among a broad range of Gram-negative bacteria^10–12^. BamB is a component of the outer membrane protein assembly complex (β-barrel-assembly machinery [Bam])^13,14^, whereas TolC is a part of the AcrAB-TolC efflux pump complex that transports various compounds with low specificity^15^. As they have distinct functions, each deletion could show different effects on membrane permeability; thus, a combined effect is expected. Although the dysfunction of each gene leads to an increase in foreign compound uptake, membrane flux properties and combined effects have not been analyzed in detail. In particular, relationships between flux changes driven by each mutation and the hydrophobicity or scaffold size of foreign compounds are still largely unknown although such information is a key factor to understand and engineer the nature of foreign compound uptake by Gram-negative bacteria. In the sensitizing approach, we used cell-penetrating peptide (CPP) conjugates of peptide nucleic acid (PNA, a nucleic acid analog) as sensitizers to silence gene expression because of their generally low toxicity and easy-to-design nature^16^. We also investigated the applicability of these approaches in the screening of antibiotic compounds and the bioconversion of value-added compounds. Furthermore, we used the sensitizing approach to *Pseudomonas aeruginosa*, another Gram-negative pathogenic bacterium, to demonstrate the potential of sensitizers for broad application in Gram-negative bacteria.

## Results

### Influx and efflux properties of the deletion mutants

*bamB* (Δ*bamB*), *tolC* (Δ*tolC*), and *bamB* and *tolC* (Δ*bamB*Δ*tolC*) deletion mutants of *E. coli* were created. *N*-Phenyl-1-naphthylamine (NPN), a probe compound, was used to investigate the influx and efflux properties of the mutants. It is a small hydrophobic fluorescent dye (molecular weight, 219; 1-octanol/water partition coefficient [log *P*_ow_], 4.2 [https://pubchem.ncbi.nlm.nih.gov]) that exhibits high fluorescence in hydrophobic environments, such as lipid bilayers of cell membranes^17^.

**Figure 1:**
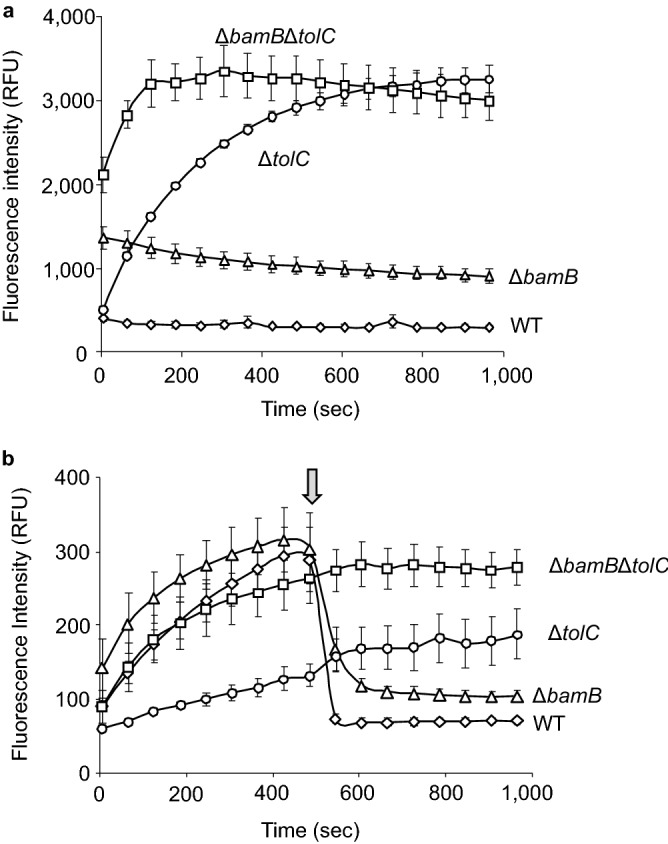
NPN influx and efflux analysis. (**a**) Result of the influx analysis; fluorescence intensity of NPN is presented as a function of time. The data are presented as the mean ± standard error of mean from triplicate experiments. (**b**) Efflux analysis. The arrow indicates the time of glucose addition. NPN, N-phenyl-1-naphthylamine; WT, wild-type strain; Δ*bamB*, outer membrane lipoprotein deletion mutant; Δ*tolC*, outer membrane efflux protein deletion mutant; Δ*bamB*Δ*tolC*, *bamB* and *tolC* double deletion mutant.

The influx properties of the wild-type strain and the mutants were analyzed by adding NPN to the cultures and monitoring fluorescence over time (Fig. 1a). In this experimental setup, an increase in fluorescence was interpreted as an increase in the influx rate or a decrease in the efflux rate. Fluorescence increased in Δ*bamB* even at time zero. This result showed that the uptake in Δ*bamB* occurred too fast to monitor a gradual increase in fluorescence. In Δ*bamB*Δ*tolC*, fluorescence also immediately increased although it gradually further increased. The fluorescence of Δ*tolC* at time zero was the same as that of the wild-type strain, and it increased gradually over time. These results suggested that influx was increased by *bamB* deletion but not by *tolC* deletion; *tolC* deletion might cause a decrease in the efflux rate.

An efflux analysis was performed using NPN and carbonyl cyanide 3-chlorophenylhydrazone (CCCP) that inhibits the proton motive force (PMF) and hence the function of efflux pumps. The cells subjected to CCCP treatment in the absence of glucose, which do not have any PMF driving efflux pumps, were resuspended in buffer with NPN, and fluorescence transition was monitored (Fig. 1b). Because of a lack of PMF, fluorescence intensity was gradually increased solely by the influx in all strains. After the addition of glucose that restored PMF and efflux flow, the fluorescence intensity immediately deceased in Δ*bamB* and the wild-type strain but not in Δ*tolC* and Δ*bamB*Δ*tolC*. This result indicated that *tolC* deletion affected efflux property. Therefore, *bamB* and *tolC* deletion resulted in an increase in influx and a decrease in efflux properties, respectively.

### Scaffold size-dependent effect of antibiotics on uptake efficiency in the deletion mutants

**Figure 2:**
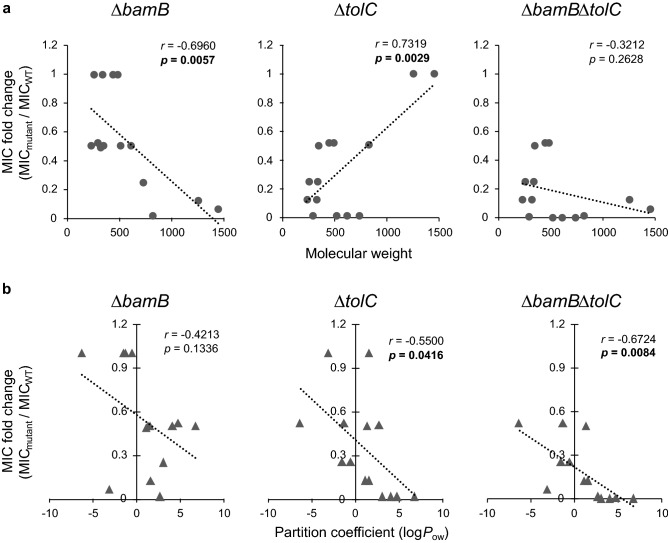
Correlation between MIC fold change and scaffold size or hydrophobicity. The MIC fold change (each mutant/the wild type strain) and (**a**) scaffold size or (**b**) hydrophobicity of antimicrobial compounds are plotted for each mutant. The dotted line represents a regression line. The correlation coefficient (*r*) and *p* value by Pearson’s correlation analysis are indicated within each panel; *p* value in bold means significant at *p* = 0.05.

The uptake efficiency of foreign compounds in the deletion mutants was further evaluated by determining the MIC of various antibiotics (Table 1). Regarding large-scaffold antibiotics, *bamB* deletion effectively improved susceptibility: the MICs of vancomycin and actinomycin D decreased by 16- and 8-fold, respectively. Conversely, small-scaffold hydrophobic antibiotics (chloramphenicol, triclosan, and 5-ketoclomazone) were more efficiently taken up by the *tolC* mutant than those by the other mutants (8-, 64-, and over 4-fold, respectively). An inverse trend of scaffold size dependency of MIC fold change (mutant/wild-type) was observed between Δ*bamB* and Δ*tolC* with statistical significance (Fig. 2a). Conversely, no clear correlation was observed in Δ*bamB*Δ*tolC*. *bamB* or *tolC* deletion likely decreased the MIC by improving the inefficient uptake of antibiotics by the wild-type strain due to a low influx or high efflux rate. The uptake of moderate-sized scaffold hydrophobic antibiotics (rifampicin, erythromycin, novobiocin, and fusidic acid) was improved via both deletions (64-, 256-, 256- and 512-fold, respectively). These results suggested that the effect of each deletion on MIC depended on the scaffold size of antibiotics. The hydrophobicity dependency of MIC fold change was commonly observed in the mutants (Fig. 2b), indicating that compounds with high hydrophobicity were more susceptible to influx and efflux changes by *bamB* or *tolC* deletion than compounds with low hydrophobicity.

**Table 1:** MIC of antibiotics for deletion mutants.

Antibiotics (molecular weight; log *P*_ow_)	Wild-type MIC (μg/mL)	Δ*bamB* MIC (μg/mL)	Δ*tolC* MIC (μg/mL)	Δ*bamB*Δ*tolC* MIC (μg/mL)
Vancomycin (1450; − 3.1)	100	6.3	100	6.3
Actinomycin D (1255; 1.6)	200	25	200	25
Rifampicin (823; 2.7)	7.5	0.12	3.8	0.12
Erythromycin (734; 3.06)	50	12.5	0.78	0.20
Novobiocin (613; 4.1)	100	50	1.6	0.39
Fusidic acid (517; 6.75)	400	200	6.3	0.78
Kanamycin (485; − 6.3)	2.5	2.5	1.3	1.3
Tetracycline (444; − 1.3)	0.25	0.25	0.13	0.13
Ampicillin (349; 1.35)	20	10	10	10
Berberine (336; − 1.5)	> 167	> 167	42	42
Chloramphenicol (323; 1.14)	4.3	2.1	0.54	0.54
Triclosan (290; 4.76)	0.25	0.13	0.0039	0.0019
5-Ketoclomazone (254; − 0.54)	> 250	> 250	63	63
Nalidixic acid (232; 1.59)	5	2.5	0.63	0.63

MIC, minimum inhibitory concentration; Δ*bamB*, outer membrane lipoprotein deletion mutant; Δ*tolC*, outer membrane efflux protein deletion mutant; Δ*bamB*Δ*tolC*, *bamB* and *tolC* double deletion mutant. Log *P*_ow_ values were derived from the PubChem database (https://pubchem.ncbi.nlm.nih.gov/) except for fusidic acid (The European Chemicals Agency, https://echa.europa.eu/registration-dossier/-/registered-dossier/20132/4/8.), berberine^18^, and 5-ketoclomazone (https://www.hpc-standards.com/msds/681800_Ketoclomazone_Acetonitrile_1_MSDS_EN_HPC-Standards.pdf).

*bamB* and *tolC* deletions elicited combined effects on the uptake of some antibiotics (erythromycin, novobiocin, fusidic acid, and triclosan; Table 1). Therefore, interactions between *bamB* and *tolC* deletions were investigated by using a fractional inhibitory concentration index (FICI), which is used to evaluate interactions (synergism, no interaction, or antagonism) between two inhibitory agents (Supplementary Table S1). The results showed that two deletions worked synergistically in moderate-sized scaffold antibiotics showing a combined effect. Moreover, triclosan, a small-scaffold antibiotic with a combined effect, also had an FICI of 0.502, which is close to the criterion for synergism (FICI ≤ 0.5).

### Uptake efficiency of CPP-PNAs by the deletion mutants

**Figure 3:**
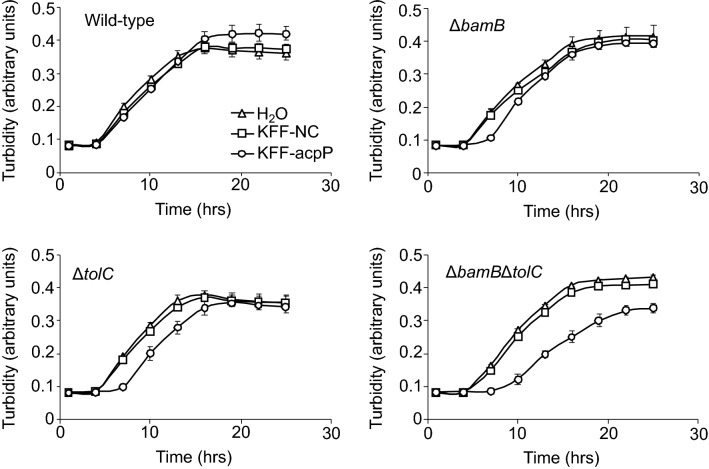
Growth of the wild-type and mutant strains in the presence of KFF-acpP. Cell growth in the presence or absence of CPP-PNAs is indicated. The CPP-PNAs used were KFF-NC (□) and KFF-acpP (○). In a control experiment, distilled water was added instead of CPP-PNAs (△). The final CPP-PNA concentration was 0.3 μM. Data are presented as the mean ± standard error of the mean from triplicate experiments. KFF-NC, negative control; KFF-acpP, CPP-PNA targeting acyl carrier protein.

Antisense CPP-PNAs are considered new anti-infective or antimicrobial drugs. PNA is a charge-neutral oligonucleotide analog that possesses good hybridization properties and resistance to degradation by nucleases and proteases^19^. The uptake efficiency of PNA alone by bacteria is usually poor; thus, CPPs such as KFFKFFKFFK are frequently conjugated to PNAs^19^. Therefore, we evaluated whether the uptake efficiency of KFF-acpP, a CPP-PNA, also increased in the deletion mutants. *acpP* is essential for growth, and KFF-acpP has bactericidal effects^19^. Subinhibitory KFF-acpP concentrations (0.3 μM) caused a slight growth delay in the single deletion mutants (Δ*bamB* and Δ*tolC*) and a relatively strong growth delay in the double deletion mutant (Δ*bamB*Δ*tolC*). However, it did not affect the growth curve of the wild-type strain, indicating that KFF-acpP was taken up more efficiently in the mutants than in the wild-type strain (Fig. 3). The differential effect of KFF-acpP on growth between the mutants and the wild-type strain was also supported by the decrease in MIC in the mutants (Supplementary Table S2).

### Proof-of-concept usages: detection of antibiotic compounds at low concentrations

**Figure 4:**
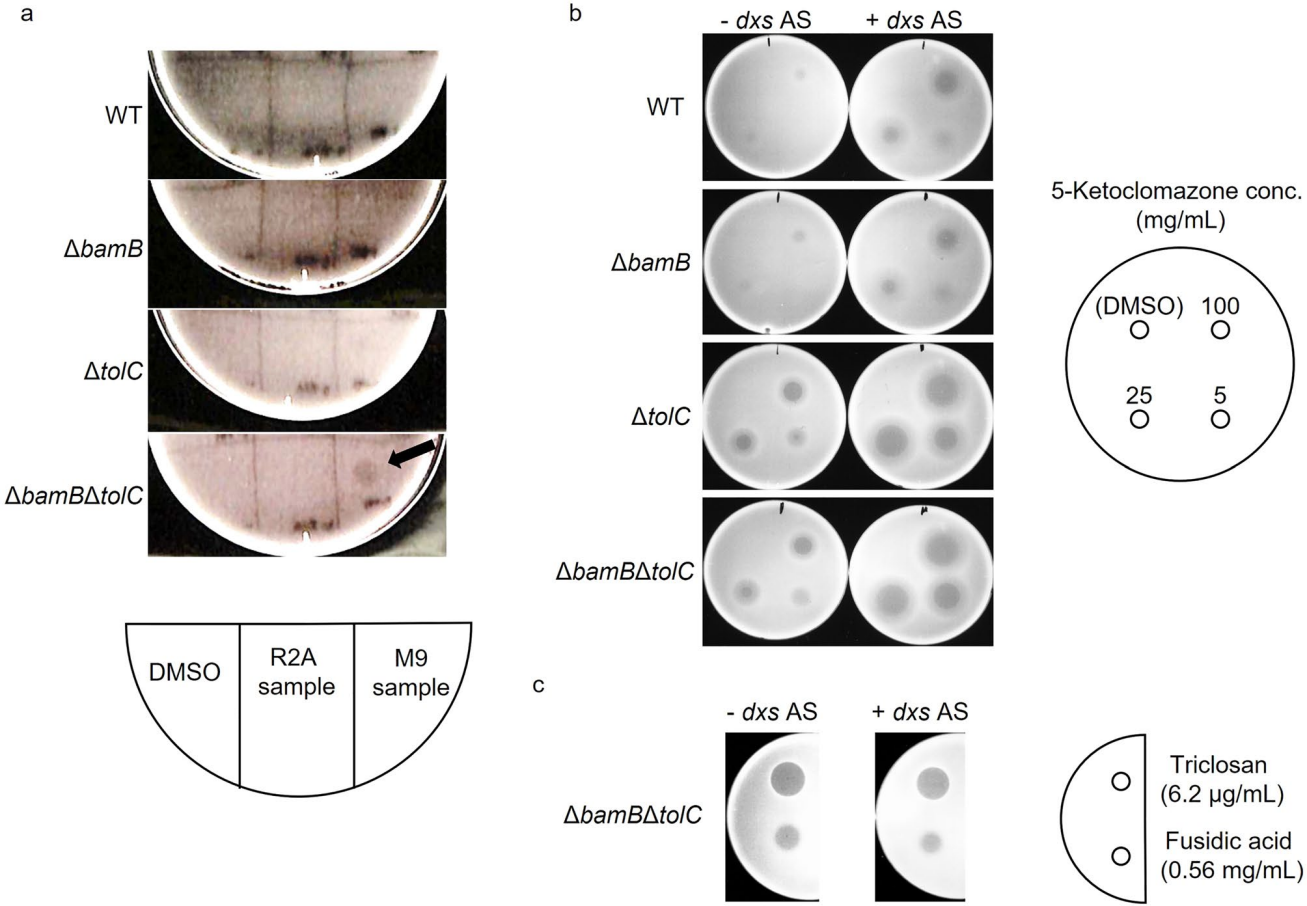
Detection of antibiotic activity in the culture samples of natural soil and gene expression titration assay. (**a**) Aliquots of the extract from soil culture with R2A (R2A sample) and M9 (M9 sample) media and control (DMSO) were spotted on the lawn of indicator strains on an agar plate as indicated by a diagram. After incubation, antibiotic activity was detected as the inhibition zone formed on the plate. The arrow indicates a halo. (**b**) 5-Ketoclomazone was spotted on the lawn of indicator strains on an agar plate at concentrations indicated by a diagram. (**c**) Control experiment was performed with triclosan and fusidic acid as test antibiotic compounds. Δ*bamB*, outer membrane lipoprotein deletion mutant; Δ*tolC*, outer membrane efflux protein deletion mutant; Δ*bamB*Δ*tolC*, *bamB* and *tolC* double deletion mutant; *dxs* AS, expression of dxs antisense RNA.

In screening novel antibiotics from natural samples, antibiotic concentrations in samples are generally low^20^. Therefore, the presence of antibiotic compounds in such samples is often difficult to be detected; consequently, sensitized strains are preferable as indicator strains. On this basis, we tested the sensitivity of the present mutants to the extracts of environmental soil samples as follows: potential antibiotic-containing samples were prepared by inoculating a soil sample with a liquid medium and extracting the culture with ethyl acetate; drops of the extracts were placed onto agar plates containing the wild-type or mutant cells. We found halos (zones of inhibition) only in the plates with Δ*bamB*Δ*tolC* cells (Fig. 4a). Therefore, Δ*bamB*Δ*tolC* was more sensitive to antibacterial compounds than the wild-type strain and could be used for detecting active compounds at low concentrations.

### Proof-of-concept usages: gene expression titration assay (GETA)

A GETA can also be used to increase the sensitivity of cells to antibiotics at low concentrations^21^. In GETA, an antisense agent is used to partially silence the expression of a specific mRNA, whose translation product is a target for a certain antibiotic; consequently, host cells become sensitized because antibiotics can elicit their effects at low concentrations.

5-Ketoclomazone was used as a test antibiotic here because it showed a low uptake by the wild-type strain (Table 1). *dxs* encoding 1-deoxy-d-xylulose 5-phosphate synthase, the putative cellular target molecule of 5-ketoclomazone^22^, was silenced by the endogenous expression of a short antisense RNA against *dxs* by using an expression vector^23^. The cells became more susceptible to 5-ketoclomazone when the antisense RNA against *dxs* was expressed (Fig. 4b), and this effect was greater in Δ*tolC* and Δ*bamB*Δ*tolC* than in the wild-type strain and Δ*bamB* even at low 5-ketoclomazone concentrations. In the negative control experiment involving antibiotics unrelated to Dxs (triclosan and fusidic acid), *dxs* silencing did not change the sizes of the halo (Fig. 4c). This result indicated that *dxs* silencing specifically increased the susceptibility to 5-ketoclomazone. Therefore, target gene silencing conferred the higher susceptibility of *tolC* deletion mutants to 5-ketoclomazone than that of the wild-type strain, and the present *bamB*/*tolC* mutants could be useful high-sensitive host strains for GETA.

### Proof-of-concept usages: conversion of inactive VD3 to active hydroxylated VD3 in the mutants

**Figure 5:**
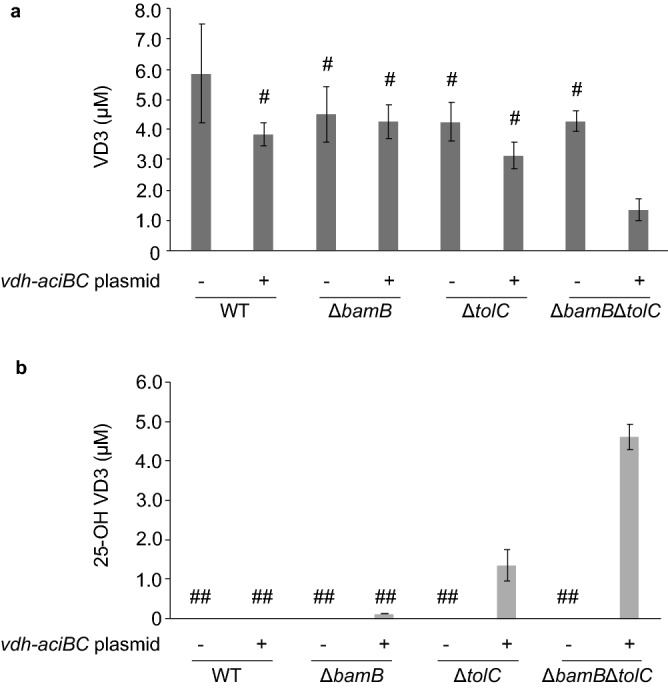
VD3 conversion efficiency of the mutants. Inactive VD3 was converted to active VD3 by using cells transformed with pHN1387 (empty vector control; *vdh-aciBC* plasmid−) or pHN4136 (*vdh-aciBC* expression plasmid; *vdh-aciBC* plasmid+). The host cells used are indicated at the bottom of the graph. The quantification result of inactive VD3 that remained after the conversion reaction from triplicate experiments is presented as the mean ± standard error of the mean. Data were statistically analyzed using ANOVA, and significant differences were evaluated with Tukey’s multiple comparison test. One-way ANOVA showed a significant difference at *F* = 2.7 (*p* = 0.046). The bars marked with “#” correspond with the groups that did not differ significantly from each other at adjusted *p* < 0.05 in the post hoc test. The unmarked bars correspond with the group that significantly differed from the other groups. (**b**) The quantification result of active VD3 produced after the conversion reaction is shown as in (**a**). VD3, vitamin D3; Δ*bamB*, outer membrane lipoprotein deletion mutant; Δ*tolC*, outer membrane efflux protein deletion mutant; Δ*bamB*Δ*tolC*, *bamB* and *tolC* double deletion mutant. One-way ANOVA showed a significant difference at *F* = 82 (*p* = 2.5 × 10^–11^). “##” is marked as in (**a**).

VD3 is a hydrophobic vitamin that contains a steroid skeleton^24^. Several forms of VD3 exist; cholecalciferol (molecular weight, 385; log *P*_ow_, 7.5 [https://pubchem.ncbi.nlm.nih.gov]) is an inactive form of VD3, and its hydroxylated forms 25-hydroxycholecarciferol (calcifediol) and 1,25-dihydroxycholecalciferol (calcitriol) are active forms of VD3 (showing a hormonal activity) and valuable as pharmaceuticals^24^. An enzymatic conversion of inactive to active VD3 in microbes is desirable because an enzymatic hydroxylation approach is more cost-effective than chemical synthesis^25^. However, a low VD3 uptake rate in *E. coli* retards enzymatic conversion^8^. Therefore, cells expressing VD3 hydroxylase (*vdh*), ferredoxin (*aciB*), and ferredoxin reductase (*aciC*) genes were prepared to improve the conversion of VD3. When the wild-type strain was used as an expression host, no VD3 conversion occurred, whereas in Δ*tolC* and Δ*bamB*Δ*tolC*, an effective conversion (1.4 and 4.6 μM, respectively) was detected (Fig. 5). Therefore, the conversion rate of inactive VD3 was improved by *tolC* deletion and further enhanced by double deletion with *bamB*.

### Sensitizing wild-type *E. coli* with anti-*bamB* and *tolC* CPP-PNAs

The aforementioned results depended on gene recombination techniques; thus, the generated mutant strains cannot be readily used in pharmaceutical and food industries because of restrictions and regulations for the use of genetically modified organisms. Instead of using gene recombinant mutants, we tested whether CPP-PNAs targeting *bamB* and *tolC* could function as efficient sensitizers for increasing the uptake of several antibiotics. Before CPP-PNAs as sensitizers were evaluated, their effect on the growth of the wild-type strain in the absence of antibiotics was assessed. Low CPP-PNA concentrations marginally affected growth, but it caused a slight growth delay at high concentrations, indicating a dose-dependent negative effect of CPP-PNAs on *E. coli* growth (Supplementary Fig. S1). The wild-type strain was then treated with antibiotics in the presence of the following CPP-PNAs: KFF-NC, KFF-bamB, and KFF-tolC (Table 2). Unexpectedly, the treatment with KFF-NC decreased the MIC of certain antibiotics, namely, actinomycin D, novobiocin, and fusidic acid (compared with the results in Table 1); this result indicated that the KFFKFFKFFK peptide fragment affected the uptake efficiency of some antibiotics. Nevertheless, KFF-bamB and KFF-tolC more effectively decreased the MICs than KFF-NC did. For example, KFF-bamB decreased the MICs of actinomycin D, novobiocin, and fusidic acid by 2-, 16-, and 4-fold, respectively; KFF-tolC decreased the MIC of 5-ketoclomazone by 2-fold. Furthermore, the combination of KFF-bamB and KFF-tolC decreased the MIC of the tested antibiotics largely than the single use of each CPP-PNA except for the MIC of vancomycin, which was not decreased by any CPP-PNAs. The interaction between KFF-bamB and KFF-tolC was evaluated by using FICI (Supplementary Table S3), and two CPP-PNAs were found to work synergistically (FICI ≤ 0.5) for actinomycin D and fusidic acid. These results indicated that KFF-bamB and KFF-tolC are effective sensitizers for *E. coli* against some antibiotic compounds.

**Table 2:** MIC of antibiotics for *E. coli* and *P. aeruginosa* in the presence of CPP-PNAs.

*E. coli*					
Antibiotics	KFF-NC MIC (μg/mL)	KFF-bamB MIC (μg/mL)	KFF-tolC MIC (μg/mL)	KFF-bamB KFF-tolC MIC (μg/mL)^a^	
Vancomycin	100	> 200	100	100	
Actinomycin D	50	25	50	< 6.3	
Novobiocin	12.5	0.8	12.5	< 0.4	
Fusidic acid	50	12.5	50	< 0.4	
5-Ketoclomazone	> 250	> 250	125	62.5	

*P. aeruginosa*					
Antibiotics	Control (no CPP-PNA) MIC (μg/mL)	RXR-NC MIC (μg/mL)	RXR-bamB MIC (μg/mL)	RXR-oprM MIC (μg/mL)	RXR-bamB RXR-oprM MIC (μg/mL)^b^
Vancomycin	1024	1024	256	512	256
Erythromycin	128	128	32	64	< 16
Carbenicillin	64	64	8	32	< 4

CPP-PNAs were added at the concentration of 5 and 3 μM for *E. coli* and *P. aeruginosa*, respectively.

^a^In the presence of both CPP-PNAs, equimolar KFF-bamB and KFF-tolC were mixed.

^b^In the presence of both CPP-PNAs, equimolar RXR-bamB and RXR-oprM were mixed.

### Sensitizing *Pseudomonas aeruginosa* with anti-*bamB* and *oprM* CPP-PNAs

To explore the applicability of CPP-PNA approach, we tested the effect of CPP-PNAs on antibiotic susceptibility in a notorious opportunistic pathogen, *P. aeruginosa*. We additionally created CPP-PNAs that targeted *bamB* and *oprM*, a *tolC* homolog in the *P. aeruginosa* genome and evaluated the potential of these CPP-PNAs as sensitizers in the same manner as *E. coli*. CPP-PNAs negatively affected the growth of *P. aeruginosa* PAO1 in the absence of antibiotics; although the same trend was observed as in *E. coli*, the effect on the growth of *P. aeruginosa* PAO1 was stronger than that on the growth of *E. coli* (Supplementary Fig. S2). Similar to *E. coli*, *P. aeruginosa* PAO1 treated with the CPP-PNAs targeting *bamB* and *oprM* was sensitized by certain antibiotics (Table 2). For all antibiotics tested, the highest MIC decrease (4-, > 8-, and > 16-fold decrease for vancomycin, erythromycin, and carbenicillin, respectively) was observed when both CPP-PNAs were simultaneously added although a synergistic interaction between RXR-bamB and RXR-oprM was not detected (Supplementary Table S3). Therefore, CPP-PNAs targeting *bamB* and *tolC* homologs function as efficient sensitizers for improving the uptake of various antibiotics by Gram-negative bacteria.

## Discussion

In *E. coli* and *Salmonella enterica*, the loss of BamB function leads to reduced levels of most major outer membrane proteins^11^. The resulting perturbation in outer membrane composition likely increases the outer membrane permeability. In this study, the increase in permeability mediated by *bamB* deletion was particularly effective in improving the uptake of large-scaffold compounds (LSCs; Table 1). Conversely, *tolC* deletion remarkably affected permeability for improving the uptake of moderate-sized to small-scaffold compounds (SSCs; Table 1). The present result of *tolC* deletion is consistent with a previous finding, which demonstrated that *tolC* deletion is ineffective in improving the uptake of large-scaffold antibiotics^26^; however, information about the effect of *bamB* deletion on permeability in terms of scaffold size is lacking. The observed changes in the scaffold size-dependent uptake efficiency in the mutants could be attributed to the continuous influx and efflux of SSCs and the blocking influx of LSCs by the outer membrane. The increased membrane permeability by *bamB* deletion results in an increased influx of SSCs and LSCs, but cellular SSCs could be excreted by the efflux function attributed by membrane pumps such as TolC. Therefore, as long as the efflux function is working, the cellular concentration of SSCs can be maintained at certain levels. This finding was supported by constant concentrations of NPN in the wild-type strain and Δ*bamB* (Fig. 1a). In addition, a previous study indicated that the loss of BamB function exceptionally leads to an increase in TolC abundance in the outer membrane^11,27^, thereby contributing to a slight change in MICs for small-scaffold antibiotics in Δ*bamB*. However, an increase in the influx of LSCs is not compensated by the efflux function and results in the accumulation of LSCs; for this reason, Δ*bamB* was mainly sensitized to large-scaffold antibiotics. As for Δ*tolC*, the deletion caused efflux dysfunction; consequently, SSCs accumulated, whereas the influx of LSCs were not affected. As a result, sensitivity to small-scaffold antibiotics increased. The MICs of moderate-sized scaffold antibiotics decreased in Δ*bamB* and Δ*tolC* (Table 1), indicating that moderate-sized scaffold compounds could have intermediate traffic properties compared with those of SSCs and LSCs.

Combined effects of *bamB* and *tolC* deletions were detected, and they worked synergistically in a few moderate-sized scaffold antibiotics (Supplementary Table S1). Intriguingly, these antibiotics have relatively higher hydrophobicity than the other compounds tested here, indicating that hydrophobicity is another key parameter of the influx and efflux properties of foreign compounds in *bamB* and/or *tolC* deletions. Normally, as the outer membrane of Gram-negative bacteria can work as an effective barrier for hydrophobic compounds^28^, disturbance in the outer membrane affected the traffic of hydrophobic compounds irrespective of scaffold size.

The number of antibiotics that are newly developed and approved for clinical use has decreased consistently^29^, and the Infectious Diseases Society of America has stated that the development of new antibiotics against Gram-negative bacteria showing multi-drug resistance (GNMDR) is insufficient^30^. In addition, the effects of large-scaffold or hydrophobic antibiotics on GNMDR strains have not been studied extensively^1^. Therefore, an antisense-based sensitizing approach shows potential for inhibiting the growth of GNMDR strains of *Pseudomonas*, *Acinetobacter*, *Enterobacteriaceae*, and other bacteria. In contrast to conventional low-molecular-weight sensitizer compounds whose screening and development are labor intensive, CPP-PNA sensitizers can be easily designed and synthesized according to peptide and nucleotide sequence information. Furthermore, peptide and nucleotide sequences of CPP-PNAs can be changed easily if sensitizer-resistant mutants emerge. Previous studies demonstrated that other gene silencing agents, such as peptide-conjugated phosphorodiamidate morpholino oligomers (PPMOs) that target AcrAB-TolC components, can sensitize *E. coli* and *P. aeruginosa* to some antibiotics^31,32^. Otoupal et al.^33^ implemented CPP-PNAs targeting various genes, including *tolC*, to potentiate antibiotic efficacy in *E. coli*. Previous and present results indicated the potential of CPP-PNAs as *E. coli* and *P. aeruginosa* sensitizers for the development of future therapeutic use of many antibiotics at low concentrations^34,35^. For the first time, we demonstrated not only the potential of *bamB* as a target of antisense oligonucleotide-based techniques but also the combined effect of *bamB*-targeting CPP-PNAs with those targeting *tolC* homologs. The combined effects of CPP-PNAs targeting distinct cellular processes could contribute to the reduction in the dose of sensitizers and design of sensitizer cocktails to combat various pathogens with different antimicrobial resistance mechanisms. In the present study, CPP-PNAs worked additively in many cases (Table 2), indicating their potential for combined use. However, CPP-PNAs were ineffective in some cases; for example, 5 μM CPP-PNAs efficiently worked with vancomycin for *P. aeruginosa* but not for *E. coli* (Table 2). This difference could be attributed to variations in the effective concentrations of CPP-PNAs among species, targeting genes, and antibiotics. This dose dependency can explain the discrepancy between the results of MIC tests obtained by gene deletion (complete loss of function; Table 1) and silencing (transcriptional downregulation; Table 2). The trend of scaffold-size dependency showed that Δ*bamB* and Δ*tolC* increased the permeability of LSCs and SSCs, respectively, but this trend was reversed for some compounds in CPP-PNAs (e.g., novobiocin and fusidic acid). Likewise, although the occurrence of synergism between CPP-PNAs targeting *bamB* and *tolC* homologs was detected in only some antibiotics in *E. coli* (Supplementary Table S3), precise information on the effective concentration must be obtained to evaluate the interactions between CPP-PNAs with distinct target genes. Therefore, determination of the effective concentration of CPP-PNAs for each species and for each antibiotic would be a key step for using CPP-PNAs as sensitizers.

In addition to therapeutic applications, many biotechnological fields, such as drug screening and bioproduction processes, can benefit from the proposed approach that modulates uptake efficiency. Sensitized strains can serve as a strong tool for drug screening by detecting low concentrations of bactericidal compounds in natural samples (Fig. 4). Similar gene silencing techniques have also been investigated for antibiotic dereplication during screening and antibiotic adjuvant screening^36,37^. GETA-based antibiotic screening not only increases sensitivity but also allows for target-oriented screening and investigations into novel antibiotics targeting cellular processes which were not targeted previoursly (Fig. 4)^21,23,38^. GETA involving the mutants described in this study may contribute to the discovery of antibiotics with distinct modes of action from known antimicrobials. As the present mutants showed a higher uptake efficiency for an antisense agent (Fig. 3), they could be used as host cells to perform GETAs with higher sensitivity than the wild-type strain. The results could also be used as a basis for improving the uptake efficiency of bacteria for industrial applications. Active VD3 for industrial use is currently produced from cholesterol via chemical synthesis, which involves over 20 reaction steps and has a yield of < 1%^39^. With this ineffective production, active VD3 becomes costly. The typical price of active VD3 sold as a general-use reagent is > 100,000-fold higher than that of inactive VD3^24^. Therefore, our study (Fig. 5) may contribute to the development of an alternative cost-effective production of active VD3.

From these industrial viewpoints, it is advantageous that the growth of cells was almost unaffected after gene deletion(s) in *E. coli* (Fig. 2) because this property is important to facilitate cost-effective industrial biotechnological applications. However, the CPP-PNA treatment at high concentrations negatively affected the growth of *E. coli* (Supplementary Fig. S1), and the CPP-PNA treatment retarded the growth of *P. aeruginosa* (Supplementary Fig. S2). Therefore, in the future use of CPP-PNA approach in bioproduction, this undesirable and unexpected growth-inhibiting activity should be analyzed and reduced. However, from a therapeutic viewpoint, the observed growth defect by CPP-PNAs is interesting because it can be applied to inhibit the growth of pathogens^33^. Several studies have demonstrated the potential of silencing agents targeting growth essential genes of pathogens as an alternative to conventional antibiotics^19,40,41^. The effect of growth inhibition observed in this study was milder than that of other bactericidal agents and likely more durable against the generation of resistance. Reducing pathogen growth might indirectly contribute to pathogen eradication by the immune system.

In conclusion, we implemented two approaches, namely, gene deletion and gene silencing, to improve the cellular uptake of foreign compounds with various scaffold sizes. Thus, *E. coli* and *P. aeruginosa* became sensitized to various antimicrobial compounds, and an industrially important compound in *E. coli* could be converted. The two approaches could be useful for therapeutics and many biotechnology fields. They might be further utilized for constructing sensitive cell-based biosensors to detect environmental compounds at low concentrations (e.g., endocrine disruptor detection) or for conducting efficient bioremediation (e.g., pollutant degradation). Further analysis on the detailed mechanisms of the improved uptake (e.g., direct microscopic observation) would contribute to future applications of these two approaches in medical and biotechnological fields.

## Methods

### General genetic manipulations

*Escherichia coli* MG1655 (wild-type strain; National BioResource Project, Mishima, Japan) and its derivative strains were used in the study. All strains were cultured in Luria–Bertani broth (LB; 10 g/L Difco Bacto tryptone, 5 g/L Difco yeast extract, 10 g/L NaCl) in the presence and absence of appropriate antibiotics at 37 °C with shaking unless otherwise stated. Mueller–Hinton broth (MHB) was purchased from BD Biosciences (Detroit, MI, USA). The composition of the M9AFC medium was as follows: Na_2_HPO_4_·12H_2_O (17 g/L), KH_2_PO_4_ (3 g/L), NaCl (0.5 g/L), NH_4_Cl (1 g/L), MgSO_4_·7H_2_O (0.49 g/L), CaCl_2_·2H_2_O (0.015 g/L), FeSO_4_·7H_2_O (0.0083 g/L), thiamine-HCl (0.01 g/L), glucose (1 g/L), 5-amino levulinic acid (0.08 g/L), and casamino acid (10 g/L). Plasmids were constructed as described in “Supplementary Methods S1”, and genes were deleted in accordance with previously described methods^42^. CPP-PNAs used in this study are shown in Table 3. All CPP-PNAs were synthesized by Panagene Inc. (Daejeon, South Korea), and a stock solution (200 μM) was prepared using distilled water.

**Table 3:** List of CPP-PNAs used in this study.

CPP-PNA	Target mRNA	Sequence^c^	References
KFF-acpP	*E. coli acpP*	KFFKFFKFFK-O_linker-ctcatactct	^19^
KFF-NC	None^a^	KFFKFFKFFK-O_linker-atactaacag	This study
KFF-bamB	*E. coli bamB*	KFFKFFKFFK-O_linker-catcgggtcc	This study
KFF-tolC	*E. coli tolC*	KFFKFFKFFK-O_linker-tgcattcctt	This study
RXR-NC	None^b^	RXRRXRRXRRXRXB-ctacttatgc^d^	This study
RXR-bamB	*P. aeruginosa bamB*	RXRRXRRXRRXRXB-catatcattg	This study
RXR-oprM	*P. aeruginosa oprM*	RXRRXRRXRRXRXB-tcaggcctct	This study

CPP-PNA, cell-penetrating peptide conjugate of peptide nucleic acid; *acpP*, a gene for acyl carrier protein AcpP; *bamB*, a gene for outer membrane lipoprotein BamB; *tolC*, a gene for outer membrane efflux protein TolC; *oprM*, a gene for outer membrane protein OprM.

^a^No match with *E. coli* MG1655 genome as antisense.

^b^No match with *P. aeruginosa* PAO1 genome as antisense.

^c^Uppercase and lowercase letters indicate peptide and PNA sequences, respectively, and PNA sequences are shown from the N-terminus to the C-terminus. The O_linker portion is composed of polyethylene glycol.

^d^ X and B indicate 6-aminohexanoic acid and beta-alanine residues, respectively.

### Influx and efflux experiments using NPN

The influx and efflux rates of NPN were measured as described previously^43^. The detailed protocol is described in “Supplementary Methods S1”.

### Monitoring of *E. coli* and *P. aeruginosa* growth and measurement of MIC

Microbial cultures were prepared by diluting an overnight preculture in MHB and cultured in a well of a 96-well clear microtiter plate (Coaster, Cambridge, CA, USA; Product No. 3997). All antibiotics were tested in 2-fold serial dilutions, and the total culture volume was adjusted to 150 μL. Other conditions for MIC determination were applied as described by Muheim^1^. Water was used as the solvent for preparing stock solutions of ampicillin and kanamycin. NaOH solution (10 mM), ethanol (50%), absolute ethanol, and dimethyl sulfoxide were used to prepare nalidixic acid, tetracycline, chloramphenicol, and other antibiotics, respectively. The turbidity of the culture at 600 nm was measured using a Multiskan Sky microplate reader (Thermo Fisher Scientific, Foster City, CA, USA) every 1 h.

*Pseudomonas aeruginosa* strain PAO1 was used in this study. MIC was measured as described earlier except for the total culture volume (100 μL), incubation (Thermomixer, Eppendorf, Germany), and bacterial growth determination (visual observation). Water was used as the solvent for preparing stocks of vancomycin and carbenicillin, and ethanol (100%) was used to prepare erythromycin. RXR-bamB and RXR-oprM (Table 3) were synthesized^44,45^ and used in a similar manner to experiments involving *E. coli*. The effect of CPP-PNAs on the growth of PAO1 was assessed by culturing the strain in MHB containing CPP-PNAs (3 μM) at 37 °C with shaking; turbidity was measured using a HiTS microplate reader (Scinics Co., Tokyo, Japan). All MIC tests were repeated at least two times.

### VD3 conversion experiment

A VD3 conversion experiment and inactive and active VD3 quantification were performed as described previously^8,46^. In brief, wild-type and mutant strains were transformed with pHN4136 to express *vdh-aciBC* or with pHN1387, a control empty vector (“Supplementary Methods S1”). The transformant was then cultured in the M9AFC medium. Because VD3 is insoluble in water, partially methylated beta-cyclodextrin (2 g/L) and inactive VD3 solution (0.1 mM; stock solution dissolved in dimethyl sulfoxide) were added to the reaction buffer.

### Detection of antibiotic compounds at low concentrations

A black soil sample was collected from Fukuzumi Ogawa Park (Sapporo, Japan), and 1.5 g of soil was cultured in 20 mL of M9 (17 g/L Na_2_HPO_4_·12H_2_O, 3 g/L KH_2_PO_4_, 0.5 g/L NaCl, 1 g/L NH_4_Cl, 0.49 g/L MgSO_4_·7H_2_O, 0.015 g/L CaCl_2_·2H_2_O, 0.0083 g/L FeSO_4_·7H_2_O, 0.01 g/L thiamine-HCl, and 10 g/L glucose) or R2A medium (Becton Dickinson and Company, Detroit, MI). The sample was left at 25 °C for 7 days, and 1 mL of the soil suspension was extracted using 1 mL of ethyl acetate. The resulting supernatant was added to a new 50 mL conical tube. This supernatant was dried at room temperature overnight, and the dried sample was dissolved in 33 μL of dimethyl sulfoxide. A halo formation assay was performed on an agar plate^22^.

### Gene expression titration assay

5-Ketoclomazone was used as a test antibiotic compound because its uptake by the wild-type *E. coli* strain was low (Table 1). The cellular target molecule of 5-ketoclomazone has been considered to be 1-deoxy-d-xylulose 5-phosphate synthase (Dxs), which catalyzes the first reaction step of the methyl-d-erythritol 4-phosphate pathway^47,48^; however, it remains unclear whether Dxs is the actual target, especially in vivo^49^. As an antisense agent, a short antisense RNA against *dxs* was expressed endogenously using an expression vector^22^. In a negative control experiment, triclosan and fusidic acid, which are antibiotics unrelated to Dxs, were used. The wild-type and mutant strains were transformed with pHN4165 to express an antisense RNA against *dxs* mRNA or with pHN1257, a control empty vector (“Supplementary Methods S1”). An overnight preculture of the transformant was prepared, and 50 μL of the culture was mixed with 11 mL of autoclaved soft LB agar (1.2% agarose and 11 μL of 1 M isopropyl β-d-1-thiogalactopyranoside) and cooled to approximately 40 °C. After solidification, the antibiotic solutions (2.5 μL) were spotted on the plates at appropriate concentrations.

## Supplementary Information

Supplementary Information.

## Data Availability

No datasets were generated or analyzed in this study.
