# Supplementary material for: Scaffold size-dependent effect on the enhanced uptake of antibiotics and other compounds by Escherichia coli and Pseudomonas aeruginosa
Source: Sci Rep. 2022 Apr 4;12:5609. doi: 10.1038/s41598-022-09635-6 (PMC8980104; doi:10.1038/s41598-022-09635-6)
Supplement: Supplementary file 1 — Supplementary Information. [file 41598_2022_9635_MOESM1_ESM.pdf]

Supplementary Methods

Construction of plasmids to delete *bamB* and *tolC* genes (pHN4039 and pHN4045)

pHN1234 contained a temperature-sensitive version of plasmid replication origin (pSC101^{ts} *ori*), counter-selection marker gene encoding levansucrase (*sacB*), and a chloramphenicol resistance gene (*chl*^{r}) (Nakashima and Tamura, J Biosci Bioeng 2012 114:38-). The 5′-region of *bamB* was polymerase chain reaction (PCR)-amplified from genomic DNA of the MG1655 strain using a specific primer set (sSN4025, AGCCTGCAGGTTGATTCTGCACGCTCCGCTTCTC; sSN4026, GGAATAGAAGTTGCCAATGCCGC). The 3′-region of *bamB* was PCR-amplified similarly using a specific primer set (sSN4027, GGTAGCGGCATTGGCAACTTCTATTCCGTCGACGGCAATCGCATCTATCTGG and sSN4028, TGATCGGGATCCAGACCGTCAG). These two fragments were assembled via overlapping PCR using sSN4025 and sSN4028 primers, and the resulting fragment was treated with PstI and BamHI and cloned into the PstI and BamHI sites of pHN1234 to yield pHN4039. For deleting the *tolC* gene, pHN4045 was constructed similarly using four primers (sSN8088, GATGATGCAGCTGCAGCCATTG; sSN8089, ACTGAACCCAGAAAGGCTCAGG; sSN8090, GCCTGAGCCTTTCTGGGTTCAGTACCAGTCGTTCAGCAAACATCCGCAC; and sSN8091, TTTGGATCCGTCGCGTCGGTATATTTACCGTAAGC). These plasmids were used for deleting genomic *bamB* and *tolC* genes via the integration-resolution method (Nakashima and Miyazaki, Int J Mol Sci 2014 15:2773-).

Construction of pHN4136 plasmid to express *vdh-aciBC* genes

The DNA fragment harboring *lacI^q^* and *trc* promoter was excised from the pTrc99a plasmid (Amersham Pharmacia, Piscataway, NJ, USA) using NsiI and NcoI, and was cloned into the NsiI and NcoI sites of pHN1257 (Nakashima et al., Nucleic Acids Res 2009 37:e103) to yield pHN1387. This plasmid harbored $lacI^q$, *trc* promoter, *rrnB* terminator, $pSC101^H$ replication origin, and a kanamycin resistance gene. The pTip-QC2-vdh-aciBC plasmid harbors a *vdh* gene derived from *Pseudonocardia autotrophica* (GenBank accession number AB456955.1) and ferredoxin (*aciB*)-ferredoxin reductase (*aciC*) genes of an *Acinetobacter* sp. (GenBank accession number AB221118.1). A T107A mutation is generated in the *vdh* gene to increase enzymatic activity of VD_3 hydroxylase (Yasutake et al., Chembiochem 2013 14:2284-). The DNA fragment containing *vdh-aciBC* was PCR-amplified from pTip-QC2-vdh-aciBC using a specific primer set (sSN8219, ATGGCGCTGACCACCACCGGC; sSN8230, AAAACTAGTCTACCCCATCAACGCCTG). Before PCR amplification, sSN8219 was phosphorylated using T4 DNA kinase. The resulting fragment was treated with SpeI and purified (fragment A). To obtain a fragment containing $lacI^q$, *trc* promoter, *rrnB* terminator, $pSC101^H$ replication origin, and kanamycin resistance gene, PCR was performed using pHN1387 and the primers sSN8116 (GGTCTGTTTCCTGTGTGAAATTG) and sSN1020 (CTACTAGTTTTGGCGGATGAGAGAAGATTT). The resulting fragment was treated with SpeI and purified (fragment B). The fragments A and B were ligated using T4 DNA ligase to yield pHN4136. The plasmid pHN4136 is an isopropyl β-D-1-thiogalactopyranoside (IPTG)-inducible *vdh-aciBC* expression vector.

Construction of a plasmid to express anti-*dxs* antisense RNA pHN4165

A DNA fragment containing the anti-*dxs* antisense sequence was prepared by annealing two complementary oligonucleotides, sSN8266 (TCGAGCAATAAGTATTAATAGGCCCCTGATGAGTTTTGC) and sSN8267 (CATGGCAAAACTCATCAGGGGCCTATTAATACTTATTGC). The fragment was cloned into a NcoI-XhoI moiety of pHN1257 to yield pHN4165. The plasmid pHN4165 expressed high-efficiency hairpin-type antisense RNA against *dxs* mRNA in the presence of IPTG (Nakashima et al., Nucleic Acids Res 2006 34:e138.).

Influx and efflux experiment using N-phenyl-1-naphthylamine (NPN)6

For influx analysis, a culture was prepared by diluting 10 μL overnight pre-culture with 5 mL of fresh medium and cultured in a baffled Erlenmeyer flask (50 mL) until optical density at 600 nm (OD_{600}) reached 0.5–0.6. From the culture, an aliquot of 1.5 mL was added to a new tube, and the cells were harvested via centrifugation at 5,000 × g for 5 min and suspended in AB1 buffer (5 mM HEPES-NaOH, pH 7.2; 5 mM glucose) to adjust the OD_{600} of the suspension to 0.5. A 0.1 mL aliquot of the suspension was added to a well of a 96-well black microtiter plate (Coaster, Cambridge, CA, USA; Product No. 3915). To initiate the influx of NPN, 0.1 mL of NPN solution (20 μM in AB1 buffer) was added to the well, followed by monitoring the fluorescence emission using a SPARK 10m microplate reader (Tecan, Männedorf, Switzerland). The microplate reader was operated at 350 nm excitation wavelength, 420 nm emission wavelength, 60 gain value, 30 flashes, and interval reading of 15 s.

For efflux analysis, the cells were cultured similarly as mentioned above. A 4 mL aliquot of the culture was taken, and the cells were harvested via centrifugation at 5,000

× g for 5 min, washed with AB2 buffer (5 mM HEPES-NaOH, pH 7.2; 1 mM $MgCl_2$), and resuspended in AB2 buffer to adjust the OD_{600} of the suspension to 1.5. A 0.2 mL aliquot of the final suspension was added to a new tube, and 2 µL of CCCP solution (10 mM in dimethyl sulfoxide) was added and mixed. After the tube was left for 10 min at room temperature to inhibit the proton motive force, the cells were washed with AB2 buffer twice to remove CCCP and resuspended in 0.1 mL of AB2 buffer. The final suspension was added to a well of a 96-well black microtiter plate. Subsequently, 1 µL of NPN solution (100 mM in dimethyl sulfoxide) was added and mixed, after which fluorescence emission was monitored as mentioned above for 8 min. After 8 min, the plate was removed from the plate reader, and 5 µL of glucose (1 M) was added to the well to resume energy-dependent efflux. Measurement of fluorescence intensity was resumed immediately.

Supplementary figures and table

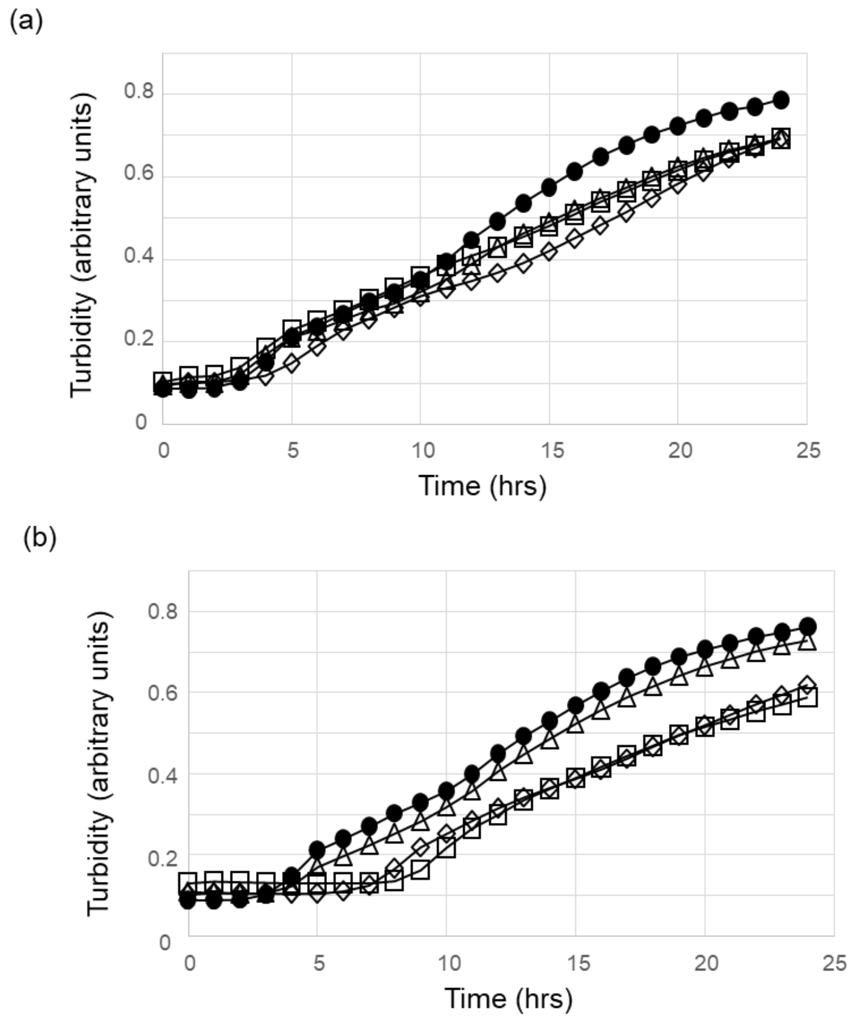

Fig. S1. Growth of wild-type *Escherichia coli* in the presence of CPP-PNAs.

A culture was prepared by diluting an overnight pre-culture in Mueller-Hinton broth and grown in a well of 96-well clear microtiter plate. Each CPP-PNA was added at (**a**) 5 µM or (**b**) 7.5 µM concentration and total culture volume was adjusted to 150 µL. The CPP-PNAs used were KFF-NC (△), KFF-bamB (□), and KFF-tolC (◇). In a control experiment, distilled water was added instead of CPP-PNAs (●). Turbidity at 600 nm

was measured every 1 h. CPP-PNA, cell-penetrating peptide conjugate of peptide nucleic acid.

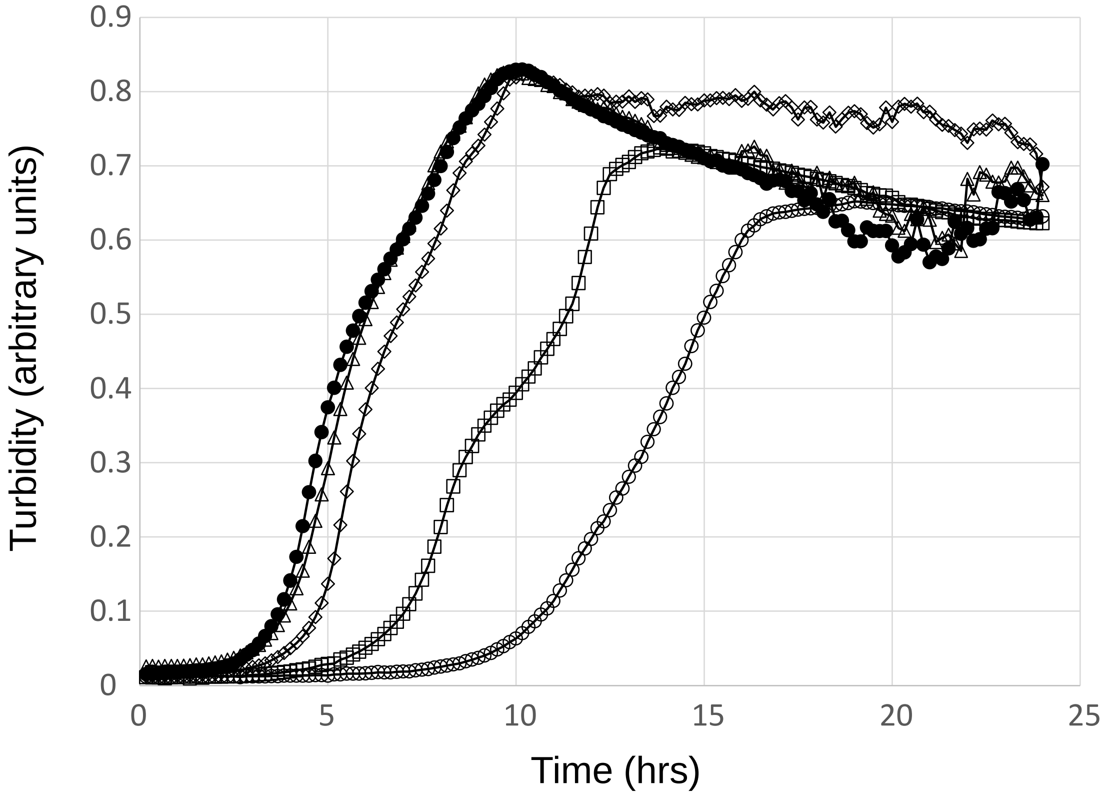

Fig. S2. Growth of *Pseudomonas aeruginosa* PAO1 in the presence of CPP-PNAs

A culture was prepared by diluting an overnight pre-culture in Mueller-Hinton broth and cultured in a well of a 96-well clear microtiter plate. Each CPP-PNA was added at 3 μM concentration, and total culture volume was adjusted to 100 μL. The CPP-PNAs used were RXR-NC (△), RXR-bamB (□), RXR-oprM (◇), and equimolar mixture of RXR-bamB and RXR-oprM (○). In a control experiment, distilled water was added instead of CPP-PNAs (●). Turbidity at 600 nm was measured every 10 min. CPP-PNA, cell-penetrating peptide conjugate of peptide nucleic acid

Table S1. MIC fold changes in mutant strains and interaction between mutations.

		MIC fold change (MIC_{mutant} / MIC_{WT})					
	log P_{ow} [a]	Δ*bamB*	Δ*tolC*	Δ*bamB*Δ*tolC*	$FIC_{\Delta bamB}$ [b]	$FIC_{\Delta tolC}$ [c]	FICI[d]
Vancomycin	-3.1	0.063	1.000	0.063	1.000	0.063	1.063
Actinomycin D	1.6	0.125	1.000	0.125	1.000	0.125	1.125
Rifampicin	2.7	0.016	0.507	0.016	1.000	0.032	1.032
Erythromycin	3.06	0.250	0.016	0.004	0.016	0.256	**0.272**
Novobiocin	4.1	0.500	0.016	0.004	0.008	0.244	**0.252**
Fusidic acid	6.75	0.500	0.016	0.002	0.004	0.124	**0.128**
Kanamycin	-6.3	1.000	0.520	0.520	0.520	1.000	1.520
Tetracycline	-1.3	1.000	0.520	0.520	0.520	1.000	1.520
Ampicillin	1.35	0.500	0.500	0.500	1.000	1.000	2.000
Berberine	-1.5	1.000	0.253	0.253	0.253	1.000	1.253
Chloramphenicol	1.14	0.488	0.126	0.126	0.257	1.000	1.257
Triclosan	4.76	0.520	0.016	0.008	0.015	0.487	**0.502**
5-ketoclomazone	-0.54	1.000	0.252	0.252	0.252	1.000	1.252
Nalidixic acid	1.59	0.500	0.126	0.126	0.252	1.000	1.252

a, partition coefficient; b, a fractional inhibitory concentration in Δ*bamB*; c, a fractional inhibitory concentration in Δ*tolC*; d, a fractional inhibitory concentration index.

$$\mathrm{FIC}\Delta bamB = \frac{\mathrm{MIC}\Delta bamB\Delta tolC}{\mathrm{MIC}\Delta bamB}$$

$$\mathrm{FIC}\Delta tolC = \frac{\mathrm{MIC}\Delta bamB\Delta tolC}{\mathrm{MIC}\Delta tolC}$$

$$\mathrm{FICI} = \mathrm{FIC}\Delta bamB + \mathrm{FIC}\Delta tolC$$

FICI data was interpreted as follows: 'synergism' (FICI ≤ 0.5), 'antagonism' (FICI > 4.0), and 'no interaction' (FICI > 0.5–4.0), according to Odds, 2003 (F.C. Odds, J Antimicrob Chemother 2003 52:1).

A part of MIC fold change was calculated using minimum or maximum possible MIC values.

Table S2. MIC of KFF-acpP for the deletion mutations

CPP-PNA	Wild-type<br>MIC (μM)	Δ*bamB*<br>MIC (μM)	Δ*tolC*<br>MIC (μM)	Δ*bamB*Δ*tolC*<br>MIC (μM)
KFF-acpP	1.0	0.6	0.6	0.6

CPP-PNA, cell-penetrating peptide conjugate of peptide nucleic acid; MIC, minimum inhibitory concentration; Δ*bamB,* outer membrane lipoprotein deletion mutant; Δ*tolC,* outer membrane efflux protein deletion mutant; Δ*bamB*Δ*tolC, bamB* and *tolC* double deletion mutant.

Table S3. MIC fold changes by CPP-PNAs and interaction between CPP-PNAs.

E. coli

	MIC fold change ($MIC_{KFF\text{-}bamB\ and/or\ \text{-}tolC}$ / $MIC_{KFF\text{-}NC}$)					
	KFF-bamB	KFF-tolC	KFF-bamB KFF-tolC	$FIC_{KFF\text{-}bamB}$[a]	$FIC_{KFF\text{-}tolC}$[b]	$FICI_{KFF}$[c]
Vancomycin	2.000	1.000	1.000	1.000	0.500	1.500
Actinomycin D	0.500	1.000	0.126	0.126	0.252	**0.378**
Novobiocin	0.064	1.000	0.032	0.032	0.500	0.532
Fusidic acid	0.250	1.000	0.008	0.008	0.032	**0.040**
5-Ketoclomazone	1.000	0.500	0.250	0.500	0.250	0.750

P. aeruginosa

	MIC fold change ($MIC_{RXR\text{-}bamB\ and/or\ \text{-}oprM}$ / $MIC_{RXR\text{-}NC}$)					
	RXR-bamB	RXR-oprM	RXR-bamB RXR-oprM	$FIC_{RXR\text{-}bamB}$[d]	$FIC_{RXR\text{-}oprM}$[e]	$FICI_{RXR}$[f]
Vancomycin	0.250	0.500	0.250	1.000	0.500	1.500
Erythromycin	0.250	0.500	0.125	0.500	0.250	0.750
Carbenicillin	0.125	0.500	0.063	0.500	0.125	0.625

a, a fractional inhibitory concentration of KFF-bamB; b, a fractional inhibitory concentration of KFF-tolC; c, a fractional inhibitory concentration index for KFF-bamB and KFF-tolC; d, a fractional inhibitory concentration of RXR-bamB; e, a fractional inhibitory concentration of RXR-oprM; f, a fractional inhibitory concentration index for RXR-bamB and RXR-oprM. MIC fold change. A part of MIC fold change was calculated using minimum or maximum possible MIC values.
